# Royal Academy of Medicine

**Published:** 1889-05

**Authors:** 


### ROYAL ACADEMY OF MEDICINE.

### THE SPREAD OF TUBERCULAR DISEASE BY CONTAGION.

E. Macdowel Cosgrave, M.D., before the State Medicine Section:

I venture to lay the following remarks before the State Medicine Section, feeling that in all probability tubercular disease will rapidly assume more and more importance in preventive medicine. It is really not many years since the medical profession shared the fatalistic views still held by the public, and indeed, the very expression "preventive diseases" so freely used until lately, seemed to mark out by a rigid line certain diseases which sanitary science might be hoped to lessen, separating them from all others.

Of late years the position of tubercular disease has greatly changed, and now the prophylaxis of phthisis and the hygienic treatment of its earlier stages have assumed great importance, and it is being recognized that it is contagious, and spreads by contagion. It is hardly necessary to point out the importance of tubercular diseases. In the decade 1871-80 phthisis was given as the cause of more than one-tenth of the deaths registered in Ireland, that is to say, 103,528 out of 966,745. Tubercular diseases generally cause about one-seventh of the deaths occurring in the United Kingdom.

An important advance was made early in 1882 by Koch's discovery of apparently specific bacteria in tubercle, and by his claim to have cultivated these bacteria external to the body, and apart from tubercular matter, in suitable cultivation media, and then to have developed tuberculosis in guinea pigs and rabbits by inoculating with the organisms thus grown. It has since been generally recognized that the disease is caused by a specific morbid irritant acting on tissue of lowered vitality.

An important declaration of the opinion of medical men was elicited by the Collective Investigation Committee of the British Medical Association in 1884, in answer to a circular asking whether phthisis was contagious, and for evidence for or against; 1,078 medical men answered; of these 673 simply wrote No, without appending any explanation. The remaining 405 reported observations; of these 105 answered No, 39 were doubtful, and 261 answered Yes.

The full force of the affirmative evidence in these statistics is much greater than its numerical expression, for the question was asked to those whose minds were more or less prejudiced in favor of a negative answer. They had been brought up to look upon phthisis as non-contagious, and so their minds were not fairly fitted to recognize contagion. It is always hard to recognize an undescribed causation, and incidents which frequently follow the one or the other, are often looked upon as cause and effect until some other interdependence is pointed out.

A good example of this fallacy is the exaggerated position the heredity of phthisis occupies in the minds of medical men. This allows observers to recognize tubercular contagion between husbands and wives, and between the sick and their attendants, but not between members of the same family. The replies received by the Collective Investigation Committee illustrate this, as out of the reported observations, no less than 158 instances are given of supposed infection between husband and wife. The spread of disease from tubercular human subjects to dogs (which are difficult to infect with tuberculosis) illustrates well the contagiousness. The late M. Thuon, of Nice,[1] infected sputa of consumptives, mixed with water as a spray, daily for an hour for several weeks, into the kennels of dogs, all of which developed pulmonary tuberculosis, and Dr. Csoker, of Vienna[2] records a case of tuberculosis in a dog whose master had died of tuberculosis some time before. The results of a very important series of experiments have lately been published by Dr. Cornet.[3] He examined the dust taken from the walls of hospital wards, asylums, prisons, and private houses where phthisical patients had been. The dust was removed by a moist sponge from near the patient's bed, but from a part which could not have been soiled directly by expectoration, or contact with dirty fingers. The dust was introduced into the bodies of guinea pigs in order to test its infectiveness; 147 specimens of dust from 133 localities, and 302 guinea pigs were used in the experiments. Of the guinea pigs, 196, or exactly half died of acute infec-

---

[1] Ap. H. Bennett, M.D., *Brit. Med. Jour.*, p. 387, I., 1889.

[2] *Med. Press and Circular*, p. 260, I., 1889.

[3] Die Verbreitung der Tuburkel bacillus Ausserhalt des Korpen von Dr. George Cornet.

tion other than tuberculosis. Of those that did not die of more acute affections, the following percentages died of tuberculosis:—47·6 per cent. of those inoculated with hospital dust, 17·6 per cent. of those inoculated with asylum dust, and 43·6 per cent. of those inoculated with dust from private houses. Dr. Cornet considers that the expectoration is the source of contagion, that whilst moist it is comparatively harmless as of course bacilli cannot evaporate, but that when the excretion is dried, and becomes pulverized the danger of contagion is great.

This is Koch's[1] opinion also; he says "A phthisical patient is bound, under the circumstances in which these patients are at present generally found, to scatter around him a large quantity of infective material, and that in the form most likely to give rise to infection."

Fisher and Schill's[2] experiments throw light upon this, showing as they do that tubercle-bacilli may retain their virulence for forty-three days in putrefying sputum, and for 186 days in sputum dried at the ordinary temperature of the air.

The best proof of this power of contagion is the effect of ingestion of tuberculous matter by dogs, as they are not (like rabbits and guinea pigs) predisposed to tuberculosis, but rather antagonistic to it.

A futher advance was made when the possibility was recognized of milk from tubercular udders causing tuberculosis. A great many observers have testified to this, and Bang at the International Medical Congress, Copenhagen, stated that he had examined the milk from twenty-seven cows suffering from tubercular mammitis, and had found the specific bacilli in the milk or sediment, and produced the disease by inoculation or ingestion of the milk or sediment.

On July 9th, 1888, Dr. G. Sims Woodhead laid the results of his researches before the Grocers' Company. Having alluded to the fact that tubercular mammitis was of extreme rarity in the human female, Dr. Woodhead showed that it was of comparatively common occurrence in the cow. Out of over 600 cows Dr. Woodhead and Prof. McFadyean examined in the Edinburgh dairies they found 37 in which there was mammitis; in the milk of six of these they found tubercle-bacilli. In one of these and subsequently in five other cases they found bacilli in enormous quantities in the udder. The new tubercular tissue was disseminated in patches of various sizes throughout a portion of the gland, the new growth invaded the lobules so that a gradual transition from the healthy gland substance to the dense tubercular mass might be seen. In the mass itself the characteristic bacilli were present in almost inconceivable numbers; on careful examination of the gland, especially at the margin of the new growth, ulcerations into the ducts might be made out, and a small mass of tubercular granulations seen projecting into the lumen. In the granulation tissue, in the epithelial cells, and even lying free in the lumen there were frequently numerous bacilli, and it could easily be understood how they found their way into the milk. Bacilli could only be demonstrated in epithelial cells still attached, and also in rare cases in those lying free in the apparently healthy milk ducts. Dr. Woodhead went on to state his opinion that these facts, together with the feeding experiments recorded by so many observers, went far to prove that milk was a source of tubercular infection, especially in young children.

Prof. Bang in a paper read before the Copenhagen Medical Society on Feb. 28th, 1888, goes so far as to state that milk from cattle with pulmonary phthisis contained bacilli, even when the udders were not in any way affected, but Dr. Woodhead's observations on the rapid and insid-

[1] "Micro-Parasites in Diseases—Etiology of Tuberculosis."

[2] Ap. Koch, loc cit.

ious manner in which the udders become affected when there is tuberculosis elsewhere throw a doubt on this statement.

At a Congress held in Paris last year, the danger of the communication of tubercular disease from cows to human beings was fully recognized and an important resolution carried in the following sense:—"That every means, including the compensation of owners, should be taken to bring about the general application of the principle that all meat derived from tubercular animals, whatever the gravity of the specific lesions, should be seized and totally destroyed.

Two other important resolutions were passed by the same Congress. One recommending that dairies, etc, should be inspected to ascertain that the cows are not suffering from contagious diseases (including tuberculosis) capable of being communicated to man; the other recommending that the public should be instructed in simple means of avoiding the danger incurred by using the flesh of tubercular animals, and in proper methods of disinfecting the excreat of phthisical patients and objects contaminated by them.

The contagiousness of tubercular disease explains a great many things, the full significance of which did not before appear. For instance, it has been for long recognized that over-crowding and the re-breathing of respired air was a fruitful cause of pulmonary phthisis. In the Report of the Health of Towns' Commission, 1844, and the Army Sanitary Commission, 1858, this is clearly recognized. The latter gives the following amongst other pertinent statistics:—the Foot Guards with a cubic space per man of 331 feet had phthisis mortality of 13·8 per 1,000, whilst the Horse Guards with cubic space of 572 feet had a mortality of 7·3, or in other words, with less than double cubic space, had very little more than half the death-rate.

Contagion explains also to some extent, how tubercular disease is so likely to spread in families without having to accept heredity as such an important factor as it is generally considered. Some statistics lately published by C. Theodore Williams, M.D.,[1] are of interest in this connection.

Dealing with the improvement in tuberculosis resulting from residence in high altitudes, he gives the following table:—

| | Whole no. of cases. per cent. | | Cases with family predisposition. per cent. |
|---|---|---|---|
| Cured, greatly improved..... | 82·25 | .... | 82·25 |
| Deteriorated.... | 17·02 | .... | 16·12 |

As the improvement is just as well marked in those with a hereditary taint as in those without, it seems probable that the danger arises not from heredity as formely understood, but from the low resistance of the weakened tissues of the offspring of consumptives, and by contagion from the consumptive to his or her offspring. A possible result of the latter may be seen in family predisposition being more common amongst women than men in the proportion of 57 to 43.[2] This may partly be accounted for by contagion, female children staying more at home and being more the subject of kissing.

Another phenomenon that the contagiousness of phthisis throws light upon is that those whose family history is clear and who show no predisposition to tuberculosis are often attacked when they have been for a long time in a debilitated condition. So in text books, miscarriages, unfavorable confinements, over-lactation, etc., are named as predisposing causes. From a state medicine, as well as from a medical, point of view it is very important to recognize that tuberculosis is contagious, and that this contagion can take place in several ways:—by auto-infection, by direct contagion from lower animals, by direct contagion from the human subject, and by indirect contagion through fomites.

[1] "Medico-Chirurgical Transactions," vol. lxxxi, p. 325.

[2] "Quain's Dictionary," art. Phthisis.

The possibility of auto-infection is chiefly interesting from a medical point of view, but it gives a useful hint for treatment by antiseptics, destruction of excretæ, etc., and unlimited fresh air so as to reduce the danger of re-breathing air to its lowest possible limits. It also explains some remarkable cases. I remember the late Dr. B. G. Macdowel relating the case of a young man who had advanced phthisis pulmonalis with cavities. His case was considered hopeless and, despair making him reckless, he gave up all treatment, went to the West of Ireland, spent his days shooting over the mountains, taking no precautions against cold, wet, or over-fatigue. The result was he improved and practically recovered, living for many years afterwards.

Another somewhat similar case was published two years ago by Dr. Neal;[1] the treatment, however, was carried out under medical supervision. The patient was kept warm, fresh air was freely admitted, the windows and doors being thrown open, and antiseptic inhalations were used. The result was satisfactory, the patient rapidly improving.

These seem to have been examples of desperate struggles between cells and bacteria, in which all was staked on a single throw, and in which the cells won by cutting off the bacteria from their base of supplies. The stoppage of contagion from animals is clearly in the province of State Medicine, and this has already been recognized by some States. In France the President of the Republic last year signed a decree providing *inter alia* that every cow found to be suffering from tuberculosis shall be isolated, and that the Sanitary Inspector shall be present when it is slaughtered, and make a report on the post-mortem appearances. The meat shall be condemned if tubercular lesions are generalized. The sale of the milk of tubercular cows is absolutely prohibited, and it can only be used for feeding animals after it is boiled.

In Copenhagen the Milk Supply Company have been advised to order a fortnightly examination by a veterinary surgeon of all cows supplying milk to the Company: this examination to include a most careful search for tubercular udders. In the United Kingdom we are sadly behind. The Decree of the French Government might well be taken as a model. The recommendations of the French Congress as to dairies ought also to be adopted. The latter is very important in Dublin. With nearly four hundred dairy yards scattered through the city, frequently in a filthy condition, as graphically described by Dr. C. F. Moore in a paper read before this section,[1] as sanitarians we ought not to rest satisfied until all dairy yards are removed outside the city. The third means by which tuberculosis spreads, this is from person to person, either directly or indirectly, is also of great importance. In a paper read by the Registrar-General before this section[2] an apparent spreading of tuberculosis from crowded centres to the surrounding districts is shown. On comparing the maps of the distribution of phthisis and of bronchitis it is seen that the death-rate from bronchitis is high in Dublin and Belfast, but that the high rates scarcely spread into the districts around: whilst the phthisis high death-rate does spread into the surrounding districts. One special factor in this case is probably contagion. The young go to service in towns, become reduced in resisting power, fall ill of phthisis, return home and are shut up in small houses where they rapidly get worse and give the disease to other members of the family. As the Registrar-General pointed out small houses *per se* are not the cause, for in Ireland inferior

[1] *British Medical Journal*, 1886, vol. ii. pp. 1068.

[1] "Transactions" Academy of Medicine, 1886,
[2] "Transactions," Academy of Medicine, 1887.

house accommodation and slight prevalence of phthisis are nearly parallel, not as cause and effect but because rural life coincides with both low phthisis rate and defective nursing. The prevalence of phthisis in the rural districts surrounding large cities is therefore a noteworthy fact.

Dr. Flick[1] in a recent paper gives some interesting instances of countries where phthisis was unknown until introduced by immigrants. He alludes to the way in which in cities cases of the disease occur clustered around a moving focus.

An interesting case showing the danger of contagion to those predisposed to the desease is mentioned by Dr. Williams[2] though not with the object of illustrating this point. A young lady "with disease at the left apex, who passed one winter at Davos, lost apparently all symptoms, and nearly all signs of disease in her chest. She then married a gentleman far advanced in consumption and went to Colorado and New Mexico, where he succumbed. During his illness he was nursed entirely by her. She returned to England in the following spring with cavities in both lungs, and with intestinal ulceration, and she died in the autumn."

Dr. Henry Bennett records the case[3] of an English officer of 27, a model of health and strength, with perfect family antecedents, no phthisis or other known taint existing. He came home from New Zealand with a young wife in an advanced state of consumption. They occupied a small cabin, the windows of which were seldom or never opened during her lifetime. The voyage lasted four months and she died three weeks before the ship arrived. A few weeks after his arrival in England he developed symptoms of phthisis, and died a year or two afterwards.

[1] "The Contagiousness of Phthisis." By Laurence F. Flick, M.D.

[2] Medico-Chirurgical "Transactions," vol. 81, p. 315.

[3] *British Medical Journal* 1889, I., p. 387.

Dr. Bennett also says, "My memory recalls several cases where consumption has appeared to be thus conveyed from husband to wife, from wife to husband, from mother to child and *vice versâ*."

In connection with this a possible danger of health resorts may be pointed out. Dr. Jacoby has found tubercle bacilli in the air of an hotel in Nice in July.

Against the spread of tuberculosis sanitary science can do much. The disease should be recognized as contagious, and those affected with consumption should not be allowed to imperil the health of others. G. R. MacMullen, LL.D.,[1] suggests that steamboat companies should adopt the following rules:—

1. Intending passengers should on booking produce to the booking agent a medical certificate stating that the intending passenger is in good health, or, if not so, the nature of his or her ailment.

2. The medical officer in charge of the ship should have power to remove a passenger into the ship's hospital, which in view of such contingency, would be properly fitted for the reception of patients.

Then precautions should be taken not to treat tubercular cases in the wards with other cases, and the nurses in charge of such cases should from time to time be changed to other duties. All sputa and other excretions should be disinfected and destroyed. The bodies of animals and of human beings dying of tuberculosis should, when possible, be cremated. The non-tubercular members of the so-called "consumptive families" should be separated from those already affected. These measures, together with those recommended to check the spread of the disease from other animals, taken in conjunction with the other efforts of modern sanitation ought to do much towards checking the spread of tuberculosis by contagion.

[1] *Australian Medical Gazette* Nov. 1888.

